# Supplementary figures and images for: Predicting kinase inhibitors using bioactivity matrix derived informer sets
Source: PLoS Comput Biol. 2019 Aug 5;15(8):e1006813. doi: 10.1371/journal.pcbi.1006813 (PMC6695194; doi:10.1371/journal.pcbi.1006813)

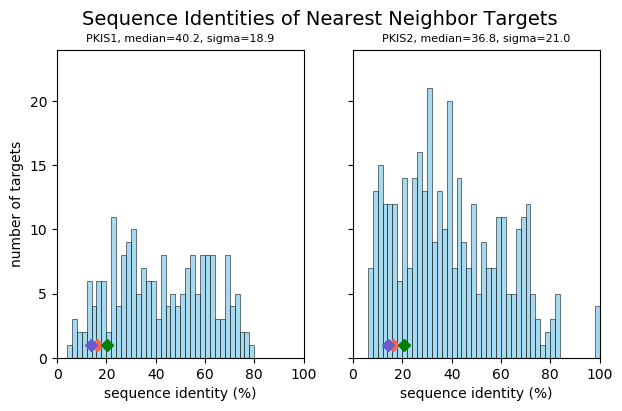

Supplement: S1 Fig — A sequence similarity matrix (% kinase domain sequence identity) was determined for most members of the PKIS1 and PKIS2 kinase sets (mutants removed). The kinase domain sequences of targets BGLF4, PknB, and ROP18 were also included. The histograms show the distribution of nearest-neighbor sequence identities among kinase domains within the matrices (PKIS1 or PKIS2). The blue (BGLF4), red (PknB), and green (ROP18) diamonds indicate nearest neighbor sequence identities observed for these targets in the PKIS1 and PKIS2 kinase sets. BGLF4, PknB, and ROP18 do not have closely related neighbors in the sets. (TIFF) [file pcbi.1006813.s001.tiff]

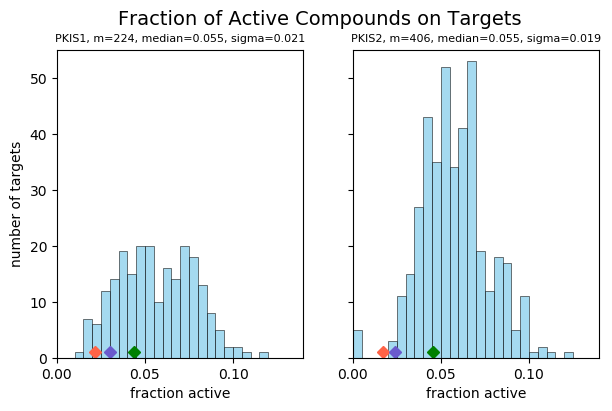

Supplement: S2 Fig — The variation in the class imbalance is even wider when a universal threshold is applied (percent inhibition) over all targets. Diamonds indicate the fraction of active compounds for BGLF4 (blue), PknB (red), and ROP18 (green) in the PKIS1 and PKIS2 compound sets. (TIFF) [file pcbi.1006813.s002.tiff]

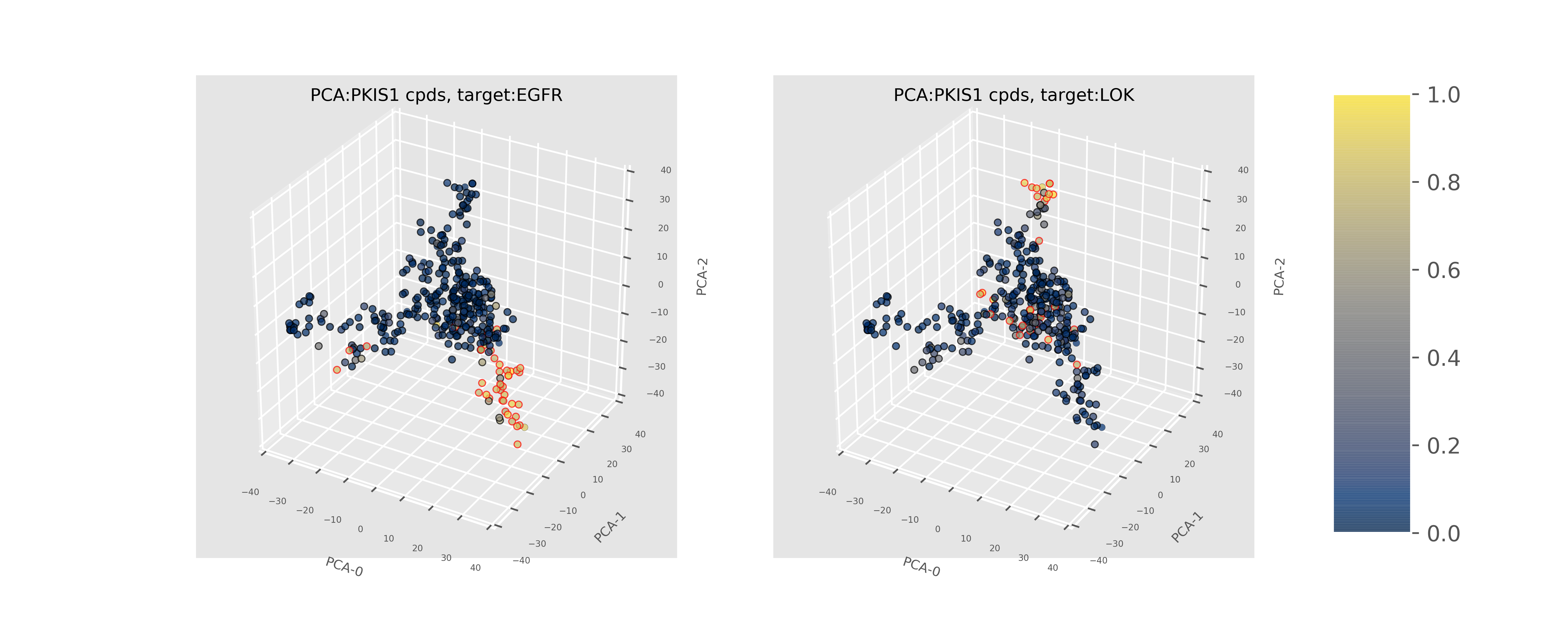

Supplement: S3 Fig — Target kinases EGFR and LOK are shown here as examples. PKIS1 compounds (points) are colored according to their experimental activity on the target: yellow indicates high activity (strong inhibition) and blue is low. Active compounds (exceeding threshold) have markers outlined in red. On these example kinase targets, separated regions of active chemical space can be observed. (TIFF) [file pcbi.1006813.s003.tiff]

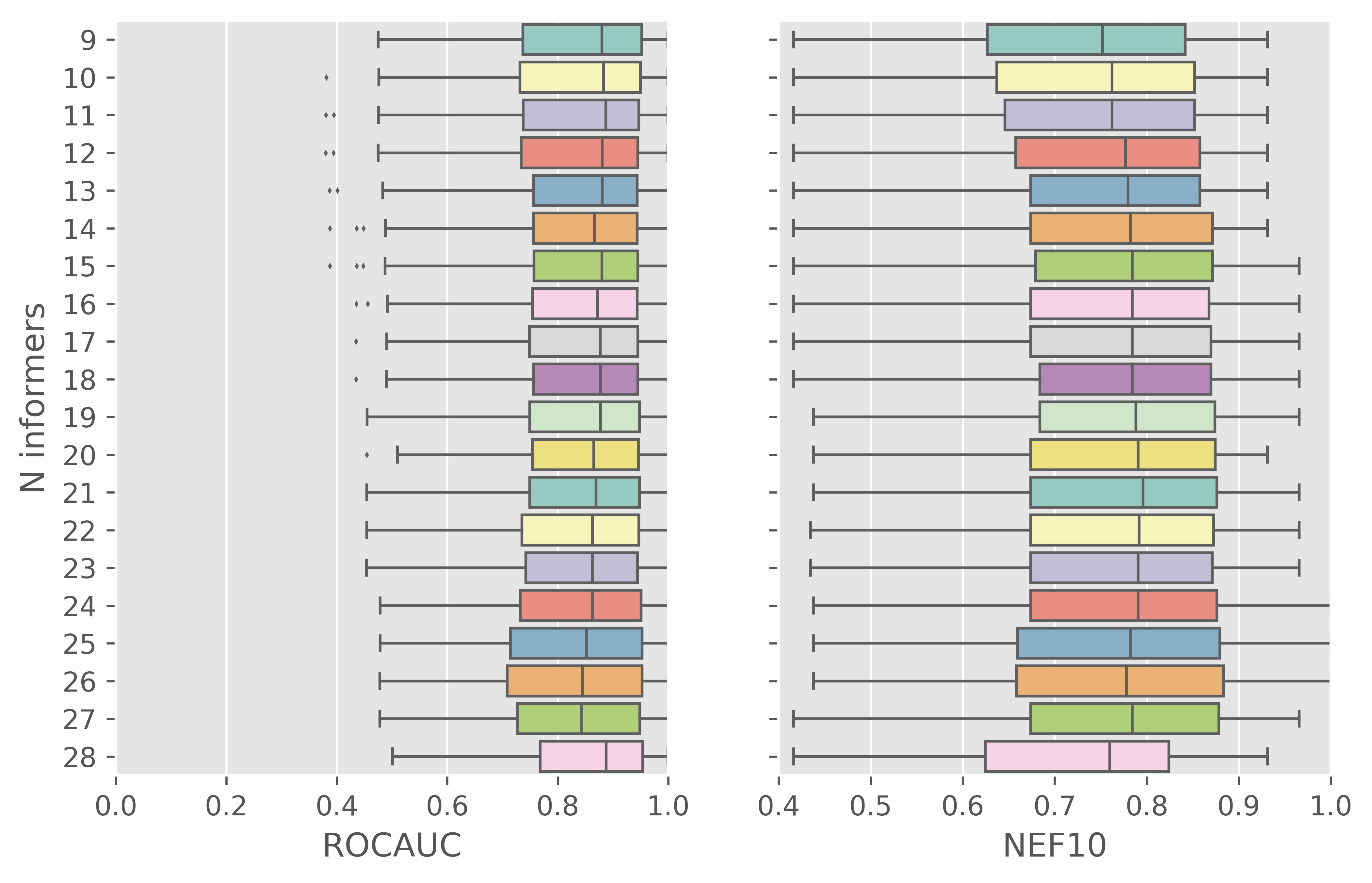

Supplement: S4 Fig — We examined the relationship between informer set size (nA = 9 to 28) for IBR method AS and virtual screening performance in terms of ROCAUC and NEF10 metrics. (TIFF) [file pcbi.1006813.s004.tiff]

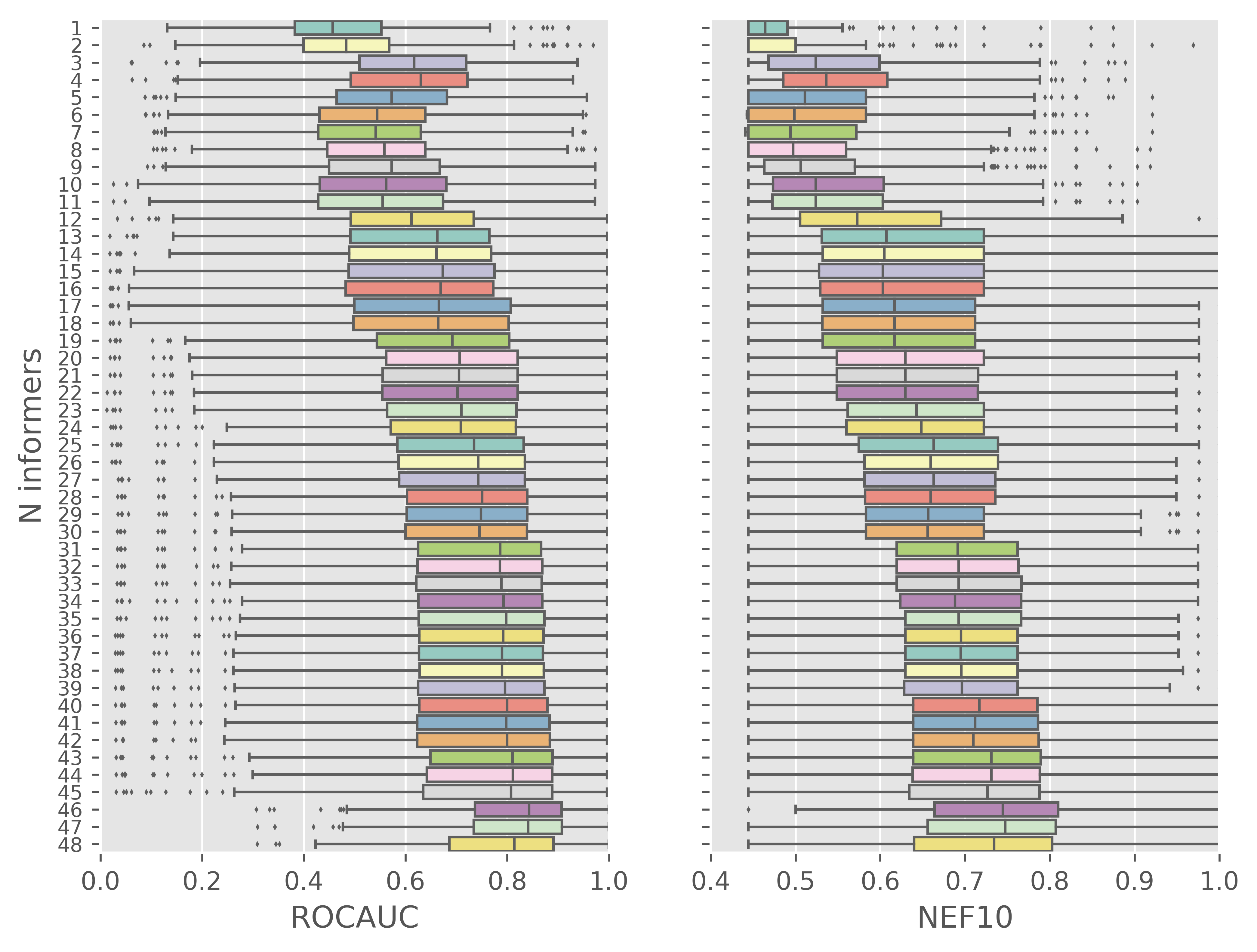

Supplement: S5 Fig — ROCAUC (left) and NEF10 (right). (TIFF) [file pcbi.1006813.s005.tiff]

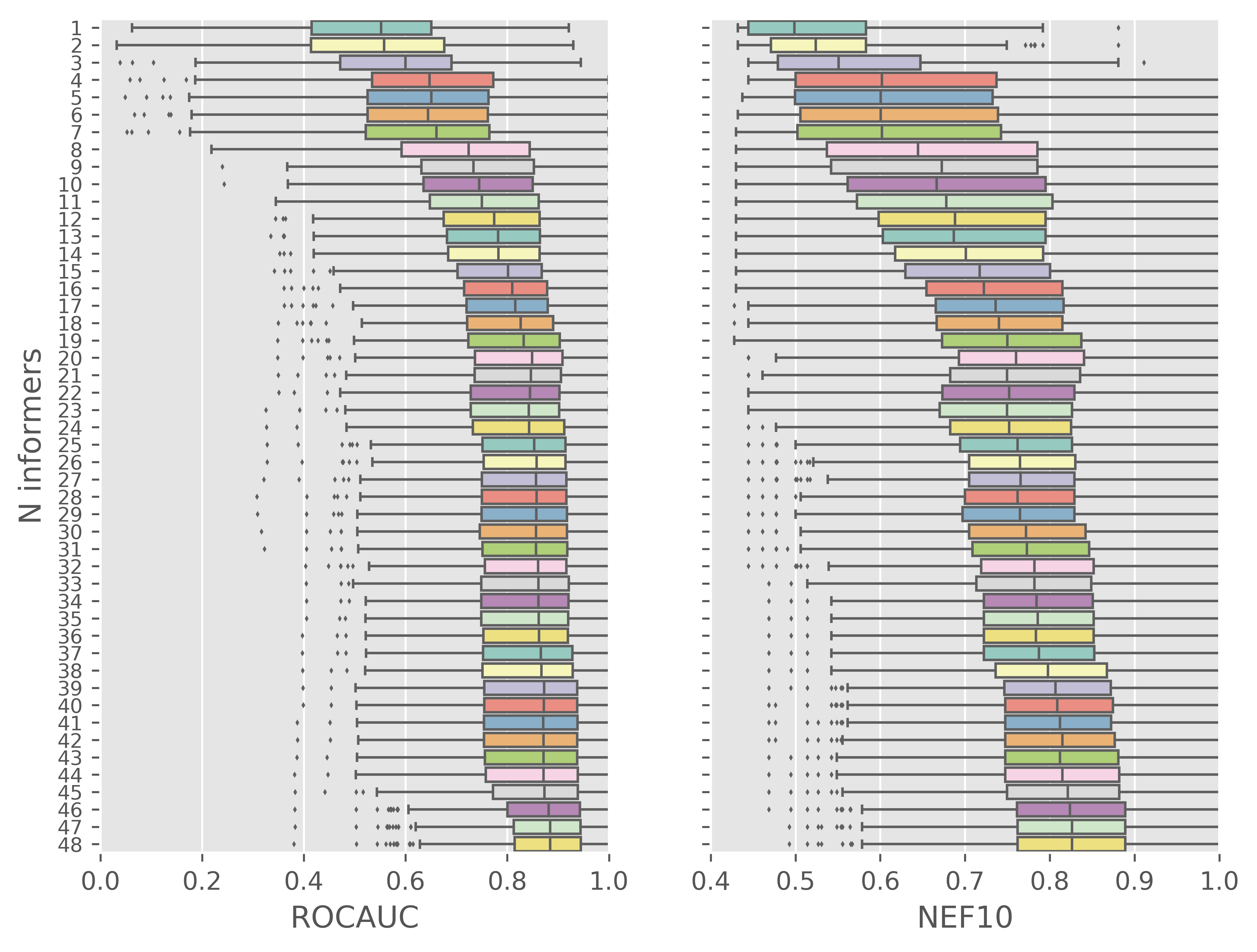

Supplement: S6 Fig — ROCAUC (left) and NEF10 (right). (TIFF) [file pcbi.1006813.s006.tiff]

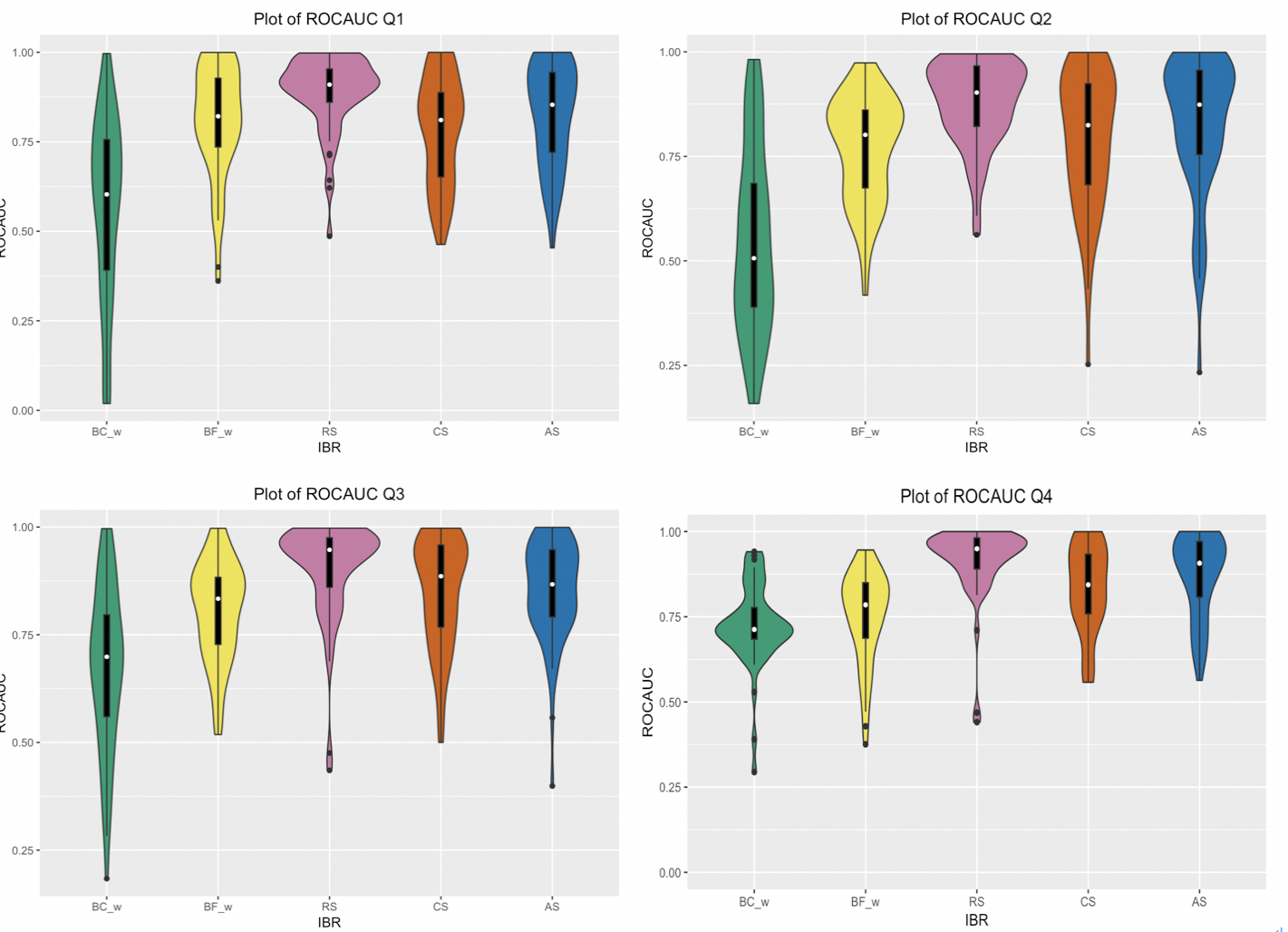

Supplement: S7 Fig — (TIFF) [file pcbi.1006813.s007.tiff]

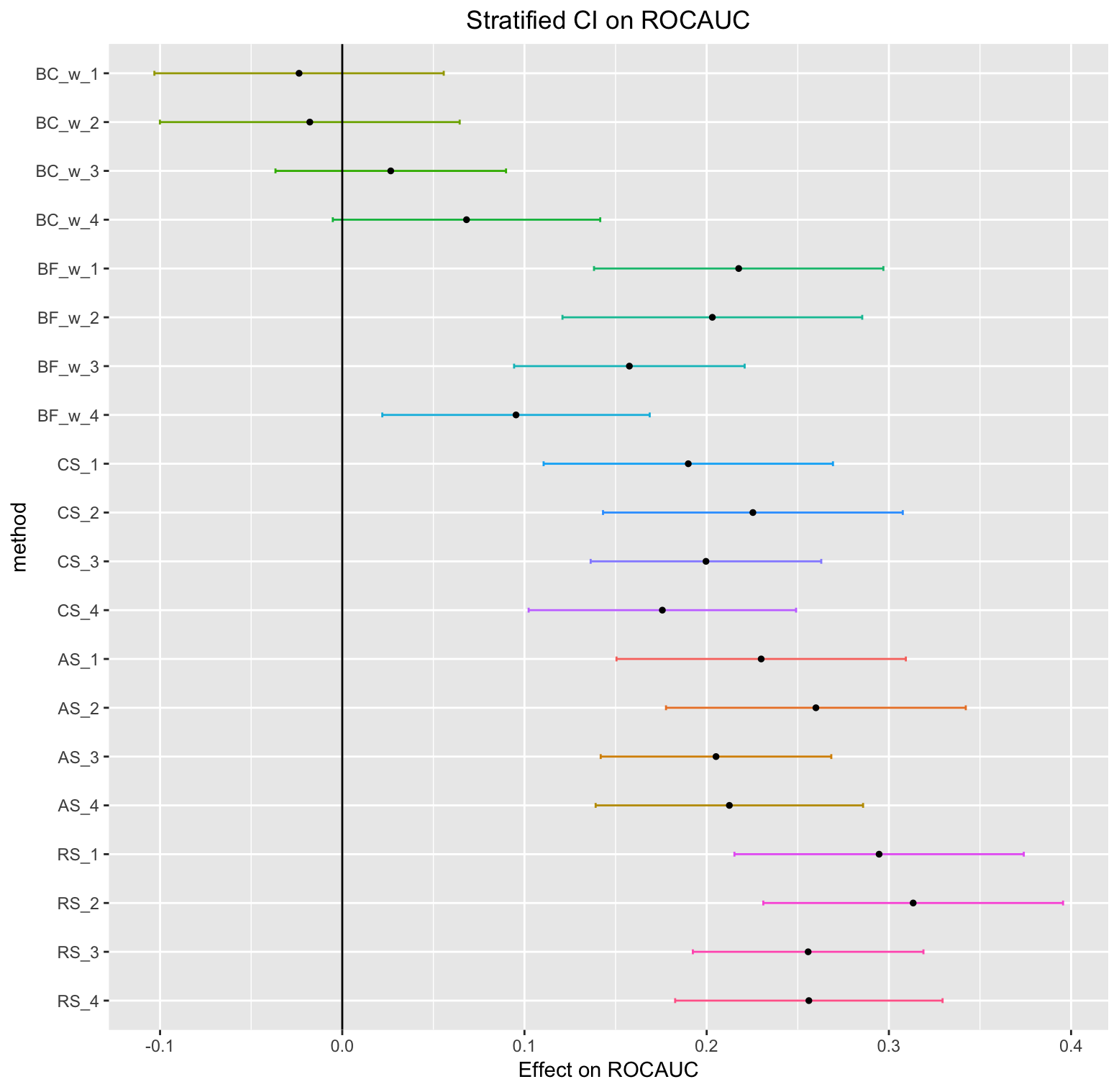

Supplement: S8 Fig — Shown are 95% confidence intervals (Tukey’s method) from a regression model allowing target and method to affect performance. (TIFF) [file pcbi.1006813.s008.tiff]

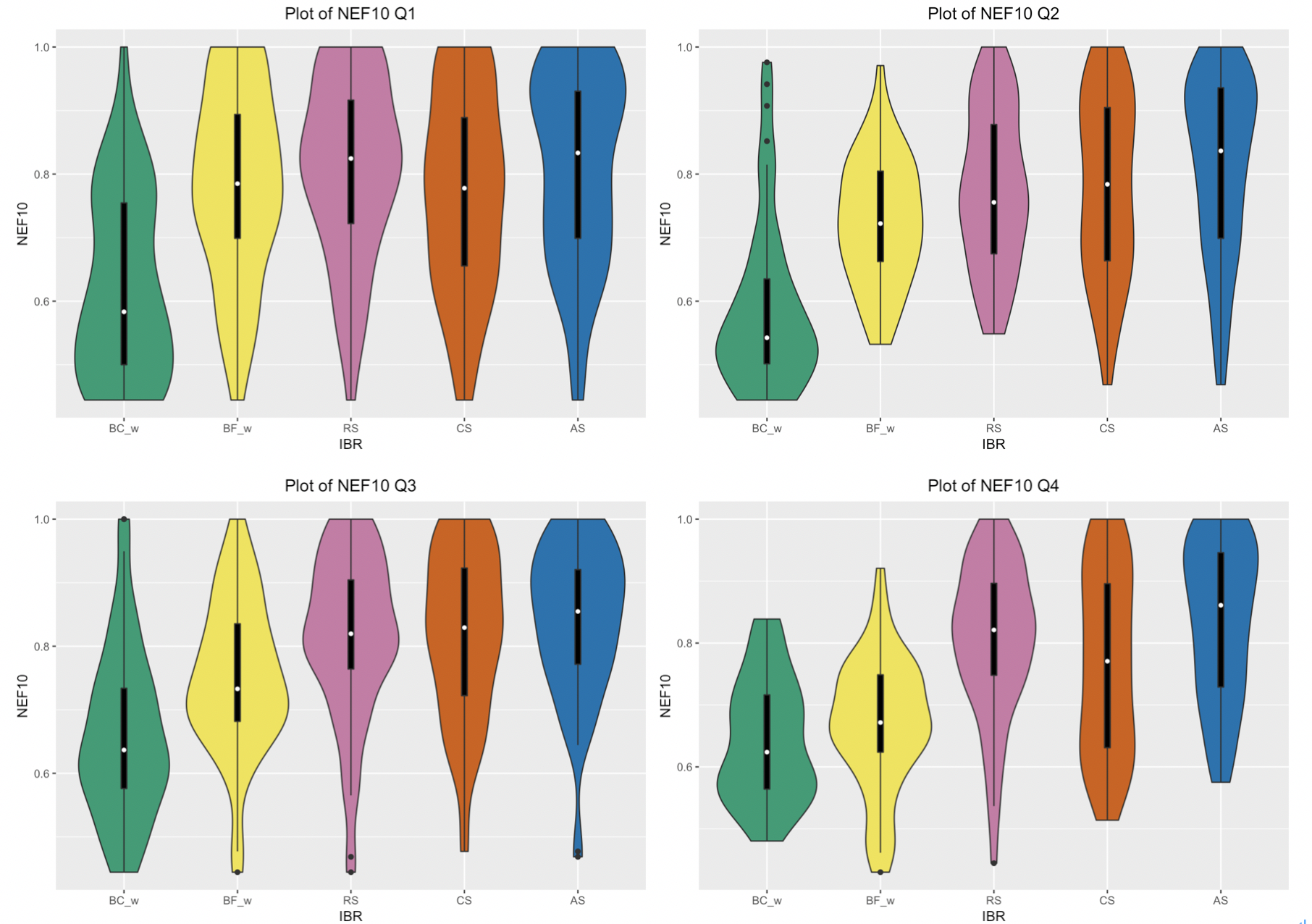

Supplement: S9 Fig — (TIFF) [file pcbi.1006813.s009.tiff]

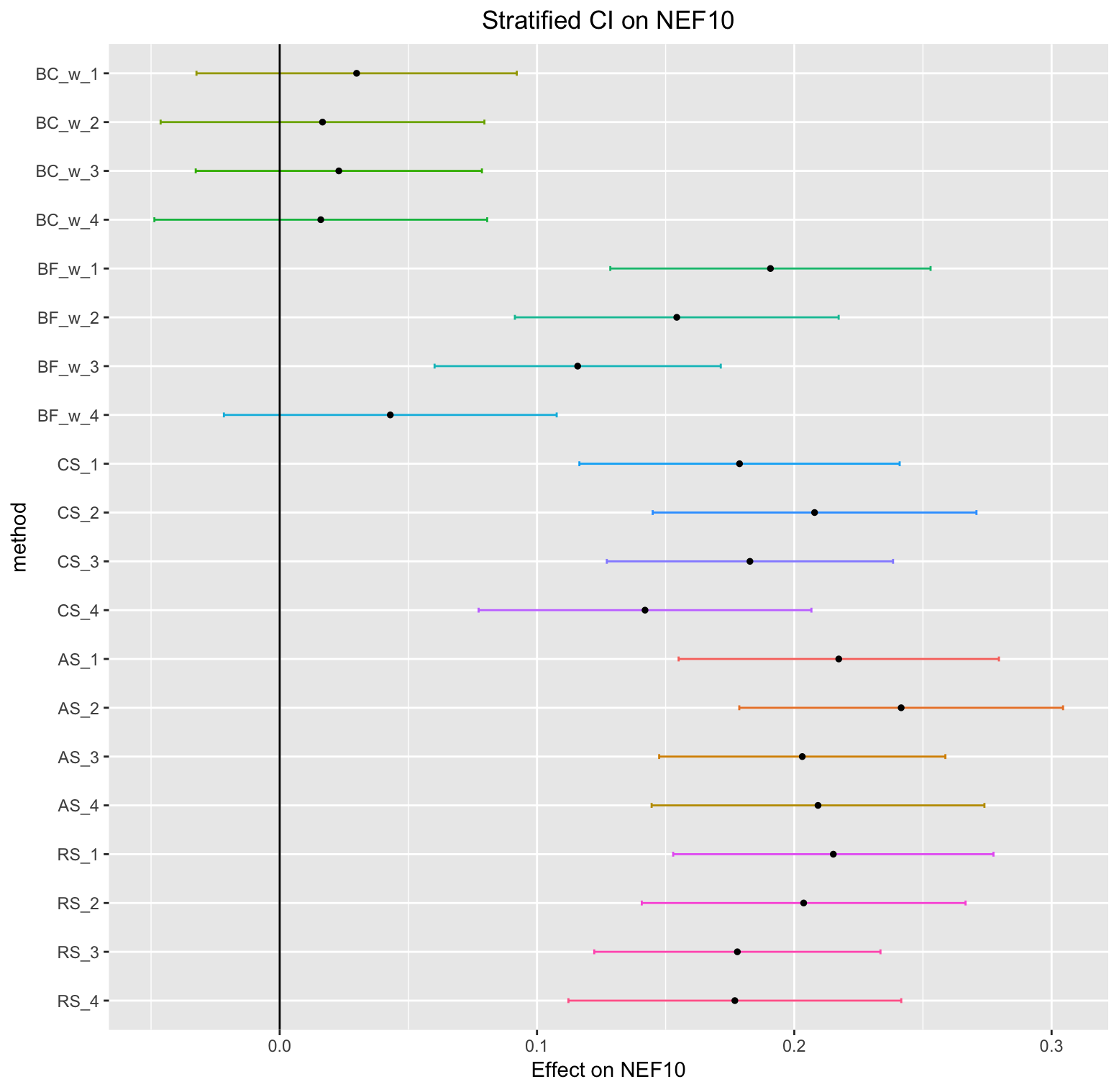

Supplement: S10 Fig — Shown are 95% confidence intervals (Tukey’s method) from a regression model allowing target and method to affect performance. (TIFF) [file pcbi.1006813.s010.tiff]

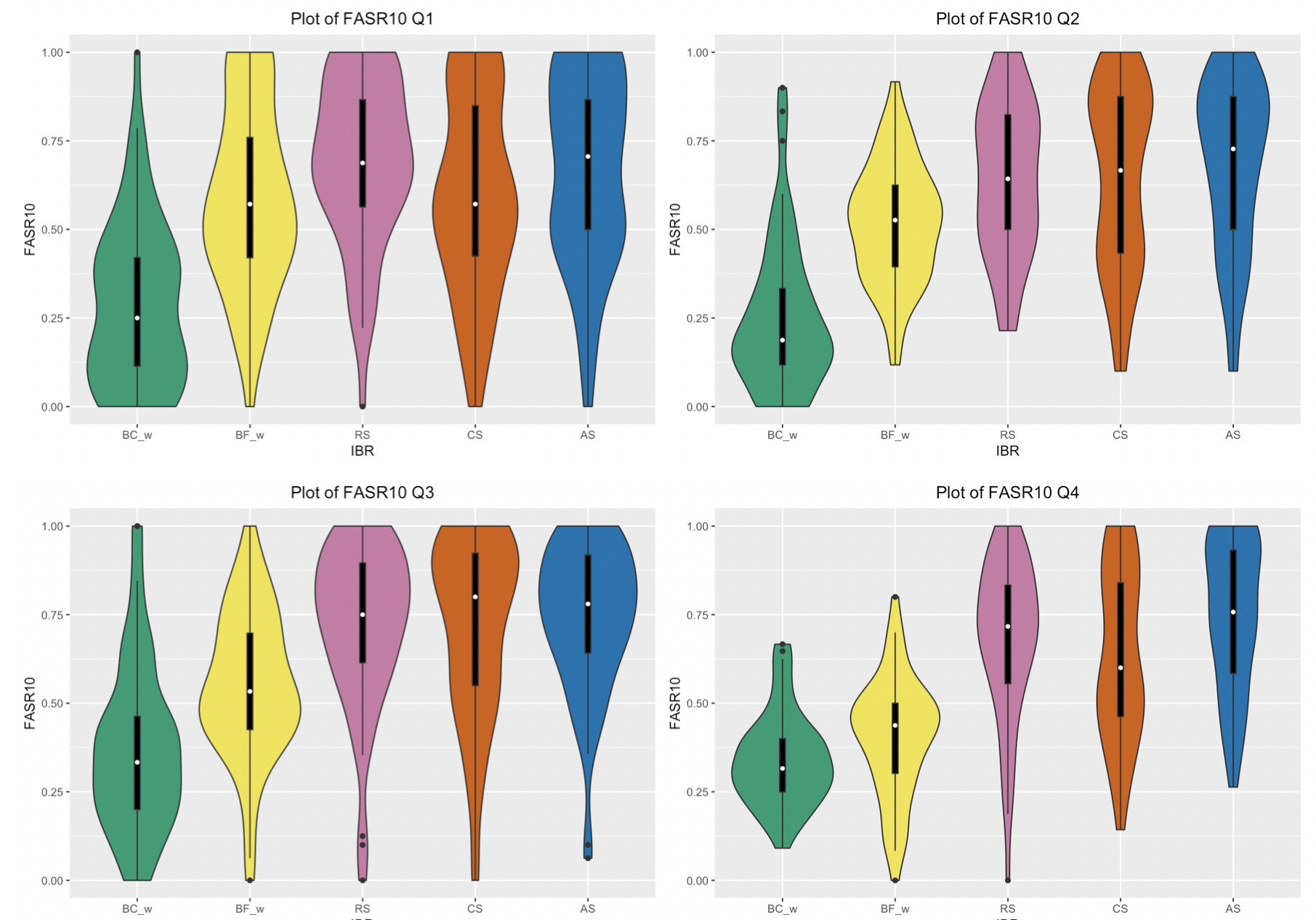

Supplement: S11 Fig — (TIFF) [file pcbi.1006813.s011.tiff]

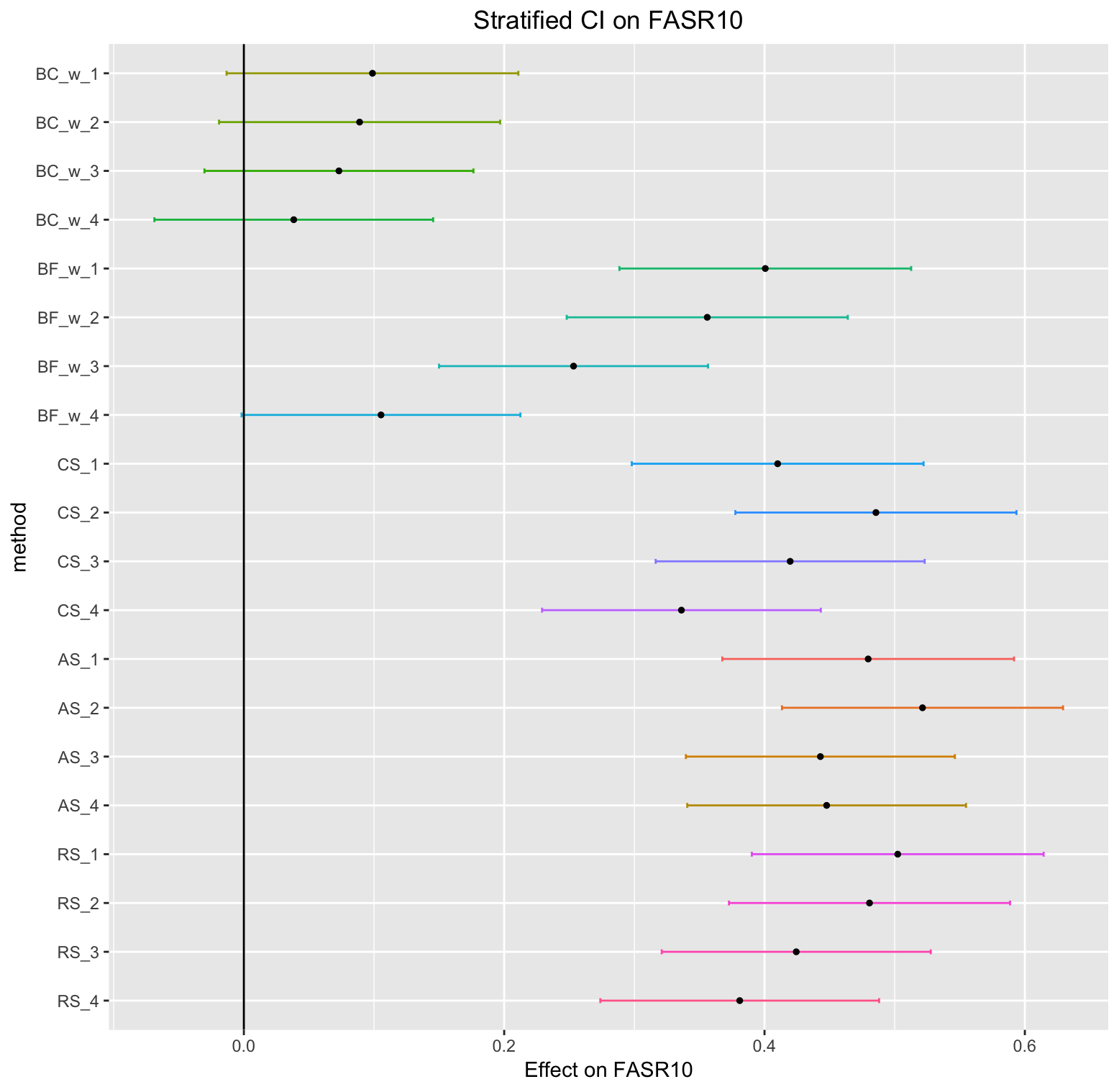

Supplement: S12 Fig — Shown are 95% confidence intervals (Tukey’s method) from a regression model allowing target and method to affect performance. (TIFF) [file pcbi.1006813.s012.tiff]
