# Supplementary material for: Predicting kinase inhibitors using bioactivity matrix derived informer sets
Source: PLoS Comput Biol. 2019 Aug 5;15(8):e1006813. doi: 10.1371/journal.pcbi.1006813 (PMC6695194; doi:10.1371/journal.pcbi.1006813)
Supplement: S1 Table — From 366 PKIS1 matrix compounds, 16 informer compounds were selected by each IBR method. The union of all informer compounds across 5 IBRs is listed. Informers selected by each method are indicated black dots. Informer activities are reported as normalized percent inhibition values [0, 1]. Informers considered active have activities reported in boldface. (PDF) [file pcbi.1006813.s013.pdf]

Table S1. Informer selections among PKIS1 compound set by IBR method.

	Activity			IBR methods				
PubChem CID	PknB	BGLF4	ROP18	BC_w	BF_w	RS	CS	AS
10173796	**0.42**	0.07	0.19		•		•	•
10308522	0.00	0.05	**1.00**		•		•	
11163861	0.11	0.15	0.05		•		•	
11281930	0.00	**0.39**	0.09			•		
11328632	0.00	0.00	0.00			•		
11626927	0.00	0.08	0.00			•	•	
11751266	0.00	0.02	0.03			•		
16048303	0.03	0.00	0.03	•				
23646938	0.01	0.07	0.00				•	•
25023715	0.00	0.02	0.00			•		•
25211574	0.00	**0.29**	0.03			•		•
25218584	0.02	0.00	0.00			•		
25218593	0.00	0.00	0.01			•		
25218600	0.00	**0.63**	0.43		•		•	•
25218601	0.00	0.00	0.18		•		•	•
25218614	0.00	**0.70**	0.07	•	•		•	
44397010	0.00	0.00	0.05	•		•		
44397250	0.04	0.00	0.06			•		
44397453	0.06	0.05	0.02			•		
44418539	0.01	0.00	0.01	•				
44532204	0.00	0.01	0.18	•				•
44532523	0.01	0.00	0.03	•				
44536036	0.01	0.00	0.04		•		•	
44581245	0.00	0.00	0.00					•
448008	0.00	0.00	0.01	•				
53239967	0.00	**0.46**	**0.85**		•		•	•
5329829	0.00	0.00	0.02		•	•		•
5329854	0.00	0.09	**0.50**	•				
5482344	0.00	**0.73**	0.01		•		•	
56604013	**0.41**	0.00	0.03					•
56604034	**0.27**	0.00	0.02		•		•	•
57391096	0.01	0.00	0.00		•	•	•	•
57396353	0.04	0.11	0.00		•			
57399798	0.01	0.02	0.04	•				
6539047	0.00	0.00	0.15	•				
6539056	0.00	0.00	**1.00**			•		
6539081	0.07	**0.33**	**1.00**			•	•	•
6539107	0.00	**0.46**	**0.85**		•		•	
6539108	0.03	**0.46**	0.10		•		•	
6539361	0.00	0.03	0.02	•				
6539593	0.00	0.00	0.03	•				
766949	0.03	0.02	0.00	•				
9604911	0.00	0.00	0.05					•
9822610	0.00	0.00	0.01		•			
9826308	0.03	0.00	0.03	•				
9901964	0.00	0.00	**0.61**	•				
9910722	0.00	0.00	0.07	•				
9927432	0.00	0.06	0.12			•		•
