# Supplementary material for: Predicting kinase inhibitors using bioactivity matrix derived informer sets
Source: PLoS Comput Biol. 2019 Aug 5;15(8):e1006813. doi: 10.1371/journal.pcbi.1006813 (PMC6695194; doi:10.1371/journal.pcbi.1006813)
Supplement: S2 Table — The values below each of the IBR methods indicate the number of active informers observed (out of 16), the number of active compounds identified in the top 10% ranking compounds by each method, and the number of distinct active scaffolds recognized in the top 10%. The total number of experimentally determined compounds and active scaffolds is indicated in the total column. For a given target, molecules included in the top 10% compounds are the active informers and the top ranking non-informers comprising 10% of the set of all compounds after removing inactive informers. (PDF) [file pcbi.1006813.s014.pdf]

Table S2. Retrieval counts by the various methods on new kinase targets (a) PknB, (b) BGLF4, and (c) ROP18 using PKIS1 or PKIS2 matrices.

(a) PknB

		baselines						non-baselines			
matrix	hits	BC_s	BC_l	BC_w	BF_s	BF_l	BF_w	RS	CS	AS	total
PKIS1	active informers	0	0	0	2	2	2	0	2	3	
	active compounds	1	1	1	7	7	7	7	2	3	8
	active scaffolds	1	1	1	7	7	7	7	2	3	8
PKIS2	active informers	0	0	0	1	1	1	0	1	1	
	active compounds	0	0	0	1	1	1	2	3	1	7
	active scaffolds	0	0	0	1	1	1	2	3	1	7

(b) BGLF4

		baselines						non-baselines			
matrix	hits	BC_s	BC_l	BC_w	BF_s	BF_l	BF_w	RS	CS	AS	total
PKIS1	active informers	1	1	1	6	6	6	3	7	4	
	active compounds	3	3	3	9	9	9	3	7	10	11
	active scaffolds	2	2	2	6	6	6	3	5	7	8
PKIS2	active informers	0	0	0	1	1	1	1	1	0	
	active compounds	2	2	1	1	1	1	8	3	1	10
	active active scaffolds	2	2	1	1	1	1	7	3	1	8

(c) ROP18

		baselines						non-baselines			
matrix	hits	BC_s	BC_l	BC_w	BF_s	BF_l	BF_w	RS	CS	AS	total
PKIS1	active informers	2	2	2	3	3	3	2	4	2	
	active compounds	4	4	4	8	8	7	4	4	2	16
	active scaffolds	3	3	3	5	4	4	2	3	2	11
PKIS2	active informers	1	1	1	0	0	0	2	0	1	
	active compounds	8	8	7	5	5	5	3	3	5	19
	active scaffolds	5	5	4	3	3	3	2	2	3	12
