# Supplementary material for: Predicting kinase inhibitors using bioactivity matrix derived informer sets
Source: PLoS Comput Biol. 2019 Aug 5;15(8):e1006813. doi: 10.1371/journal.pcbi.1006813 (PMC6695194; doi:10.1371/journal.pcbi.1006813)
Supplement: S3 Table — IBR strategies were applied prospectively on novel kinase targets, (a) Pknb, (b) BGLF4, and (c) ROP18, which do not belong to either of the PKIS1 and PKIS2 target sets. (PDF) [file pcbi.1006813.s015.pdf]

Table S3. Metrics evaluations against three new kinase targets (a) PKNB, (b) BGLF4, and (c) ROP18 using PKIS1 or PKIS2 matrices.

(a) PknB

		baselines						non-baselines		
matrix	metric	BC_s	BC_l	BC_w	BF_s	BF_l	BF_w	RS	CS	AS
PKIS1	ROCAUC	0.69	0.69	0.33	0.95	0.96	0.91	0.89	0.70	0.68
	NEF10	0.51	0.51	0.51	0.93	0.93	0.93	0.93	0.58	0.65
	FASR10	0.12	0.12	0.12	0.88	0.88	0.88	0.88	0.25	0.38
PKIS2	ROCAUC	0.56	0.56	0.32	0.47	0.47	0.45	0.79	0.51	0.65
	NEF10	0.44	0.44	0.44	0.52	0.52	0.52	0.60	0.68	0.52
	FASR10	0.00	0.00	0.00	0.14	0.14	0.14	0.29	0.43	0.14

(b) BGLF4

		baselines						non-baselines		
matrix	metric	BC_s	BC_l	BC_w	BF_s	BF_l	BF_w	RS	CS	AS
PKIS1	ROCAUC	0.38	0.38	0.35	0.94	0.96	0.89	0.74	0.78	0.94
	NEF10	0.60	0.60	0.60	0.90	0.85	0.90	0.60	0.80	0.95
	FASR10	0.25	0.25	0.25	0.75	0.75	0.75	0.38	0.62	0.88
PKIS2	ROCAUC	0.67	0.67	0.52	0.59	0.59	0.58	0.88	0.57	0.75
	NEF10	0.56	0.56	0.50	0.50	0.50	0.50	0.89	0.61	0.50
	FASR10	0.25	0.25	0.12	0.12	0.12	0.12	0.88	0.38	0.12

(c) ROP18

		baselines						non-baselines		
matrix	metric	BC_s	BC_l	BC_w	BF_s	BF_l	BF_w	RS	CS	AS
PKIS1	ROCAUC	0.42	0.42	0.43	0.84	0.79	0.78	0.76	0.55	0.44
	NEF10	0.58	0.58	0.58	0.72	0.72	0.69	0.58	0.58	0.51
	FASR10	0.27	0.27	0.27	0.45	0.36	0.36	0.18	0.27	0.18
PKIS2	ROCAUC	0.83	0.83	0.71	0.65	0.65	0.57	0.64	0.48	0.57
	NEF10	0.68	0.68	0.65	0.59	0.59	0.59	0.53	0.53	0.59
	FASR10	0.42	0.42	0.33	0.25	0.25	0.25	0.17	0.17	0.25
