# Supplementary material for: Predicting kinase inhibitors using bioactivity matrix derived informer sets
Source: PLoS Comput Biol. 2019 Aug 5;15(8):e1006813. doi: 10.1371/journal.pcbi.1006813 (PMC6695194; doi:10.1371/journal.pcbi.1006813)
Supplement: S4 Table — Nine IBR methods were evaluated on 224 PKIS1 targets using standard VS metrics that reflect active retrieval, ROCAUC, NEF10, F1, and MCC. FASR10 was also evaluated to reflect the chemical diversity of the actives retrieved. (PDF) [file pcbi.1006813.s016.pdf]

Table S4. (a) ROCAUC, (b) NEF10, (c) FASR10, (d) F_1 score (F1), and (e) Matthew's Correlation Coefficient (MCC) in Leave-One-Target-Out Cross Validation on PKIS1.

(a) ROCAUC

	baselines						non-baselines		
Methods	BC_s	BC_l	BC_w	BF_s	BF_l	BF_w	RS	CS	AS
mean	0.62	0.62	0.63	0.79	0.79	0.79	0.90	0.81	0.84
median	0.56	0.56	0.67	0.83	0.82	0.81	0.92	0.83	0.88
stdev	0.16	0.15	0.22	0.14	0.14	0.13	0.11	0.14	0.13

(b) NEF10

	baselines						non-baselines		
Methods	BC_s	BC_l	BC_w	BF_s	BF_l	BF_w	RS	CS	AS
mean	0.61	0.61	0.62	0.74	0.74	0.74	0.80	0.79	0.82
median	0.57	0.57	0.60	0.74	0.74	0.72	0.82	0.79	0.85
stdev	0.12	0.12	0.13	0.13	0.13	0.13	0.13	0.14	0.13

(c) FASR10

	baselines						non-baselines		
Methods	BC_s	BC_l	BC_w	BF_s	BF_l	BF_w	RS	CS	AS
mean	0.24	0.24	0.31	0.51	0.52	0.52	0.68	0.65	0.71
median	0.21	0.21	0.29	0.50	0.53	0.50	0.72	0.65	0.75
stdev	0.22	0.22	0.21	0.23	0.22	0.21	0.22	0.26	0.23

(d) F1

	baselines						non-baselines		
Methods	BC_s	BC_l	BC_w	BF_s	BF_l	BF_w	RS	CS	AS
mean	0.22	0.22	0.25	0.44	0.45	0.45	0.49	0.54	0.59
median	0.20	0.21	0.21	0.47	0.49	0.45	0.51	0.58	0.61
stdev	0.22	0.22	0.21	0.20	0.21	0.20	0.22	0.22	0.22

(e) MCC

	baselines						non-baselines		
Methods	BC_s	BC_l	BC_w	BF_s	BF_l	BF_w	RS	CS	AS
mean	0.19	0.20	0.21	0.41	0.42	0.42	0.47	0.52	0.57
median	0.14	0.14	0.17	0.43	0.46	0.42	0.50	0.56	0.59
stdev	0.21	0.21	0.22	0.22	0.22	0.22	0.22	0.24	0.22
