# Supplementary material for: Predicting kinase inhibitors using bioactivity matrix derived informer sets
Source: PLoS Comput Biol. 2019 Aug 5;15(8):e1006813. doi: 10.1371/journal.pcbi.1006813 (PMC6695194; doi:10.1371/journal.pcbi.1006813)
Supplement: S5 Table — VS metrics (a) ROCAUC, (b) NEF10, (c) FASR10, (d) F1 score (F1), and (e) Matthew’s Correlation Coefficient (MCC) in Leave-One-Target-Out Cross Validation on PKIS1 are compared across 9 IBR methods evaluated on 224 PKIS1 targets. A pairwise, 2-sided Wilcoxon signed-rank test (non-parametric) was applied to calculate p-values. Bold font is used to indicate p-values that fail to pass α = 0.05 threshold for significance when comparing IBR to baseline methods. To collectively compare all 6 baseline IBRs against each non-baseline IBR, we imposed a S̆idák multiple comparison correction with 6 hypotheses. This increases the stringency of the statistical threshold applied on each of the 6 individual tests to α = 0.0085. However, after applying this correction, non-baseline methods remained statistically superior to all baselines for all metrics except for CS when considering the ROCAUC metric and RS when compared on F1 and MCC metrics. (PDF) [file pcbi.1006813.s017.pdf]

Table S5. *P*-values in methods comparison for PKIS1 LOTO VS.

(a) ROCAUC

	BC_s	BC_l	BC_w	BF_s	BF_l	BF_w
RS	1.9E-31	1.6E-31	5.5E-31	1.3E-21	1.7E-21	2.7E-22
CS	3.6E-23	3.0E-23	1.0E-19	**3.2E-02**	**3.7E-02**	7.9E-03
AS	5.6E-27	3.6E-27	2.0E-23	4.9E-06	4.4E-06	7.8E-07

(b) NEF10

	BC_s	BC_l	BC_w	BF_s	BF_l	BF_w
RS	4.1E-25	1.9E-25	1.4E-24	5.9E-10	2.8E-09	1.8E-10
CS	2.1E-22	1.1E-22	1.7E-21	4.4E-07	6.6E-07	4.5E-07
AS	1.5E-28	1.9E-28	6.7E-28	1.2E-14	5.7E-14	1.3E-14

(c) FASR10

	BC_s	BC_l	BC_w	BF_s	BF_l	BF_w
RS	1.2E-30	4.5E-31	5.7E-30	2.0E-18	2.2E-17	7.5E-18
CS	4.3E-29	4.9E-29	2.1E-27	5.3E-13	3.4E-12	4.3E-12
AS	2.1E-32	2.3E-32	3.7E-32	1.2E-19	2.1E-18	9.0E-19

(d) F1

	BC_s	BC_l	BC_w	BF_s	BF_l	BF_w
RS	1.2E-11	2.8E-12	1.8E-12	**3.2E-02**	4.8E-03	**2.1E-02**
CS	4.1E-26	2.7E-27	1.1E-26	3.6E-08	3.1E-06	6.0E-07
AS	5.3E-32	1.6E-32	1.7E-32	3.4E-15	2.4E-13	1.9E-13

(e) MCC

	BC_s	BC_l	BC_w	BF_s	BF_l	BF_w
RS	6.9E-20	1.0E-20	1.3E-20	**2.2E-01**	**7.2E-01**	**4.0E-01**
CS	3.7E-25	2.0E-26	8.2E-26	4.0E-07	3.3E-05	5.4E-06
AS	1.2E-31	2.3E-32	3.4E-32	9.6E-14	4.4E-12	3.3E-12
