# Supplementary material for: Predicting kinase inhibitors using bioactivity matrix derived informer sets
Source: PLoS Comput Biol. 2019 Aug 5;15(8):e1006813. doi: 10.1371/journal.pcbi.1006813 (PMC6695194; doi:10.1371/journal.pcbi.1006813)
Supplement: S1 Text — (PDF) [file pcbi.1006813.s018.pdf]

Algorithm pseudo-code

ALGORITHM 1: Regression Selection

```
Given the bioactivity vectors for all targets, x_1, ..., x_m ∈ R^n, and the size of
  informer set n_A;
Split the data into 5 folds, each fold with roughly the same number of targets;
for K = 1, ..., 5 do
    Take the K-th fold of the data as the test data, and the rest as the training
      set;
    for j = 1, ..., n;                                              ▷ pre-processing
     do
        Linearly scale the features such that (x_i)_j for all i in the training set lie
          in the range [0, 1];
    end
    for k = 2, 3, ... do
        Cluster the training data to k categories using kmeans++ with 100
          repeats;
        Select the informer set A with n_A features by the greedy heuristic based
          on the regularized logistic regression model (3) ;
        Train a new logistic regression model (5) using the selected coordinates A;
        Use the logistic regression model to predict on the test set through (7)
          and evaluate the performance;
    end
end
Rescale the whole data set just as in the cross validation procedure;
Use the best k selected by cross validation to cluster the data;
Use the greedy heuristic to select the informer set A with size n_A;
Train the logistic regression model (5) on the whole informer set A with all
  targets;
```

ALGORITHM 2: Coding Selection

Given the binary bioactivity data $Z = \{z_{i,j}\}_{i \in I, j \in J}$;
Given a Monte Carlo sample size B;
Fix informer set size n_A;
Fix a grid $\mathcal{K}$ of cluster sizes K
for $K \in \mathcal{K}$ **do**
    for $b = 1, \ldots, B$ **do**
        Sample a set A_b uniformly at random from size-n_A subsets of J;
        Compute the code words (unique rows) of sub-matrix $Z_{A_b} = \{z_{i,j}\}_{i \in I, j \in A_b}$;
        Let L_{A_b} equal the number of code words;
        if $L_{A_b} \geq K$ **then**
            Sample a partition π_b of the code words of size K blocks, uniformly at random
        end
        if $L_{A_b} < K$ **then**
            Set π_b to be the unique partition having Ł_{A_b} blocks and constant code words within each block
        end
        Calculate

$$f_{K,\lambda}(A_b, \pi_b) = \sum_{S_k \in \pi_b} \left(\sum_{i,i' \in S_k} \left\{ 1 - \frac{\sum_{j \in A_b^c} z_{ij} z_{i'j}}{\sum_{j \in A_b^c} z_{ij} \vee z_{i'j}} \right\} \right) - \lambda L_{A_b}$$

    end
end
Rank compounds $j \in J$ by

$$f_j = \sum_{K \in \mathcal{K}} \frac{1}{B} \sum_{b=1}^{B} 1(j \in A_b) f_{K,\lambda}(A_b, \pi_b)$$

Select the best (lowest scoring) n_A compounds as the informer set.
Prioritize: Rank non-informer compounds as in Eq. (10).

ALGORITHM 3: Adaptive Selection

Input

- initial bioactivity data $X = \{x_{i,j}\}_{i \in I, j \in J}$
- a base informer set size $n_0 = 8$; final informer set size $n_A > n_0$.

Cluster targets

- Calculate a cluster number K using Eq. (12)
- Cluster targets I into K clusters using `kmeans` applied to all rows of X

Construct a base informer set A_0

- Fit a generalized linear model predicting multi-class cluster labels using R package `glmnet`, with the group LASSO penalty, and setting the penalty parameter to identify n_0 compounds that are best cluster label predictors

Adaptively expand

for *Informer sets of size increasing by one until size* n_A **do**

Add one extra compound j to A_o by Equation (13);

$$\arg\min_{j \notin A_o} \sum_{k \notin A_o \cup \{j\}} \|\boldsymbol{x}_{\cdot k} - \boldsymbol{c}_n\|_2$$

where $\boldsymbol{c}_n = \frac{1}{|A_o \cup \{j\}|} \sum_{k \in A_o \cup \{j\}} \boldsymbol{x}_{\cdot k}$.

end

Prioritize: Rank non-informer compounds as in Eq. (10).
